# Increased LIGHT expression and activation of non-canonical NF-κB are observed in gastric lesions of MyD88-deficient mice upon *Helicobacter felis* infection

**DOI:** 10.1038/s41598-019-43417-x

**Published:** 2019-05-07

**Authors:** Raquel Mejías-Luque, Ivonne Lozano-Pope, Andreas Wanisch, Matthias Heikenwälder, Markus Gerhard, Marygorret Obonyo

**Affiliations:** 1 Institute for Medical Microbiology, Immunology, and Hygiene, Technical University of Munich, School of Medicine, Munich, Germany; 2 German Centre for Infection Research (DZIF), partner site Munich, Munich, Germany; 3 Department of Medicine, School of Medicine, University of California, San Diego, La Jolla, California, USA; 4 Division of Chronic Inflammation and Cancer, German Cancer Research Center (DKFZ), Heidelberg, Germany

**Keywords:** Gastric cancer, Chronic inflammation

## Abstract

*Helicobacter pylori* infection induces a number of pro-inflammatory signaling pathways contributing to gastric inflammation and carcinogenesis. Among those, NF-κB signaling plays a pivotal role during infection and malignant transformation of the gastric epithelium. However, deficiency of the adaptor molecule myeloid differentiation primary response 88 (MyD88), which signals through NF-κB, led to an accelerated development of gastric pathology upon *H. felis* infection, but the mechanisms leading to this phenotype remained elusive. Non-canonical NF-κB signaling was shown to aggravate *H. pylori*-induced gastric inflammation via activation of the lymphotoxin β receptor (LTβR). In the present study, we explored whether the exacerbated pathology observed in MyD88-deficient (*Myd88*^−/−^) mice was associated with aberrant activation of non-canonical NF-κB. Our results indicate that, in the absence of MyD88, *H. felis* infection enhances the activation of non-canonical NF-κB that is associated with increase in *Cxcl9* and *Icam1* gene expression and CD3^+^ lymphocyte recruitment. In addition, activation of signal transducer and activator of transcription 3 (STAT3) signaling was higher in *Myd88*^−/−^ compared to wild type (WT) mice, indicating a link between MyD88 deficiency and STAT3 activation in response to *H. felis* infection. Thereby, MyD88 deficiency results in accelerated and aggravated gastric pathology induced by *Helicobacter* through activation of non-canonical NF-κB.

## Introduction

*Helicobacter pylori* is one of the most prevalent infections affecting around half of the world’s population. Inflammation elicited by *H. pylori* is characterized by strong infiltration of neutrophils, monocytes and lymphocytes into the gastric mucosa^1^. If left untreated, *H. pylori*-induced chronic inflammation can eventually progress to gastric cancer. Therefore, *H. pylori* infection is considered a main risk factor for gastric cancer and mucosa-associated lymphoid tissue (MALT) lymphoma.

Activation of a number of signaling pathways, such as nuclear factor-kappa B (NF-κB) or signal transducer and activator of transcription 3 (STAT3) signaling, in response to the infection are reported to be involved in the carcinogenetic process leading to the development of gastric tumors^2–4^.

NF-κB signaling activation is one of the first events observed in gastric epithelial cells upon *H. pylori* infection^5^. Notably, NF-κB signaling can be executed by two different arms, the canonical and the non-canonical pathways^6^, both of which have been related to *H. pylori*-induced pathology by activating the expression of several genes contributing to the inflammatory response elicited by the bacterium. Canonical NF-κB signaling is mainly activated by engagement of bacterial products to immune receptors such as Toll-like receptors (TLRs) and NOD-like receptors (NLRs)^7,8^. Signaling by most TLRs is mediated by the adaptor molecule MyD88, which was reported to confer protection from inflammation-associated cancer induced by *H. felis* infection, through yet unidentified mechanisms. Thus, *Myd88*^−/−^ mice showed a rapid progression of *H. felis*-induced inflammation to precancerous and cancerous lesions in the stomach when compared to wild type (WT) animals^9^.

Non-canonical NF-κB is activated by lymphotoxin α_1_β_2_ (LT) and LIGHT (TNFSF14; homologous to **l**ymphotoxin exhibits **i**nducible expression and competes with HSV **g**lycoprotein D for binding to **h**erpes virus entry mediator, a receptor expressed on **T** lymphocytes) produced in response to *H. pylori* infection and is dependent on a functional Type IV secretion system^10^. Binding of LT or LIGHT to LTβ receptor (LTβR) leads to NF-κB inducing kinase (NIK) activation and proteosomal processing of p100 to p52. p52 forms heterodimers with RelB that translocate into the nucleus of the cell regulating the expression of target genes involved in inflammation such as chemokines, CXCL13 and CXCL10 and cellular adhesion molecules such as vascular cell adhesion molecule (V-CAM) and intracellular adhesion molecule (I-CAM)^6,11^. Blocking of LTβR during *H. pylori* infection resulted in reduced gastric pathology, lower infiltration of inflammatory cells and decreased expression of inflammatory chemokines. These observations confirmed a direct link between LTβR signaling and *H. pylori* induced pathology *in vivo*^10^. In addition, analysis of human gastric tissue presenting with different degree of *H. pylori*-associated gastritis and gastric tumors further suggested an important role for non-canonical NF-κB signaling in gastric carcinogenesis in humans^10^.

Gastric carcinogenesis has been linked to hyperactivation of STAT3 signaling, which has been shown to be induced by *H. pylori* infection^12^. STAT3 plays key roles in embryonic development, inflammation, cell differentiation, proliferation and apoptosis as well as in tumorigenesis. Interestingly, LT produced by tumor-infiltrating B cells was reported to signal through LTβR to induce IKKα nuclear translocation and STAT3 activation^13^, while LIGHT induced STAT3 phosphorylation through NIK in prostate cancer cells^14^.

Although the role of non-canonical NF-κB signaling in *H. pylori*-induced gastritis is clear, its possible involvement in induction of gastric precancerous lesions remained elusive, since mice infected with *H. pylori* do not develop severe gastric pathology. Therefore, we assessed non-canonical NF-κB activation in gastric lesions induced by *H. felis* infection. Moreover, and given the intimate crosstalk between both NF-κB pathways during *H. pylori* infection^10^, we compared activation levels between wild type and *Myd88*^−/−^ mice, since the latter showed enhanced gastric pathology upon *H. felis* infection^9^. Our results indicate that activation of non-canonical NF-κB signaling might contribute to the early onset and rapid progression of gastric precancerous lesions observed in *H. felis*-infected *Myd88*^−/−^ mice by enhancing the expression of pro-inflammatory chemokines, favoring the recruitment of inflammatory cells and activating STAT3 in the stomach.

## Results

### Non-canonical NF-κB signaling is enhanced in *Myd88*^−/−^ mice upon H. felis infection

We previously observed that *Myd88*^−/−^ mice develop more severe pathology than WT mice upon infection with *H. felis*^9^. Thus, severe gland atrophy, hyperplasia, and dysplasia was observed in the stomach of infected *Myd88*^−/−^ mice (Supplementary Fig. 1a and ref.^9^). To identify possible molecular mechanisms involved, we first analyzed whether differences in NF-κB activation could be detected between the same previously reported WT and *Myd88*^−/−^ infected mice. To this end, we stained gastric tissue samples for p65 (Supplementary Fig. 1b). We could not detect differences between control and infected WT and *Myd88*^−/−^ mice, excluding the involvement of canonical NF-κB in more severe phenotype observed in *Myd88*^−/−^ mice.

**Figure 1:**
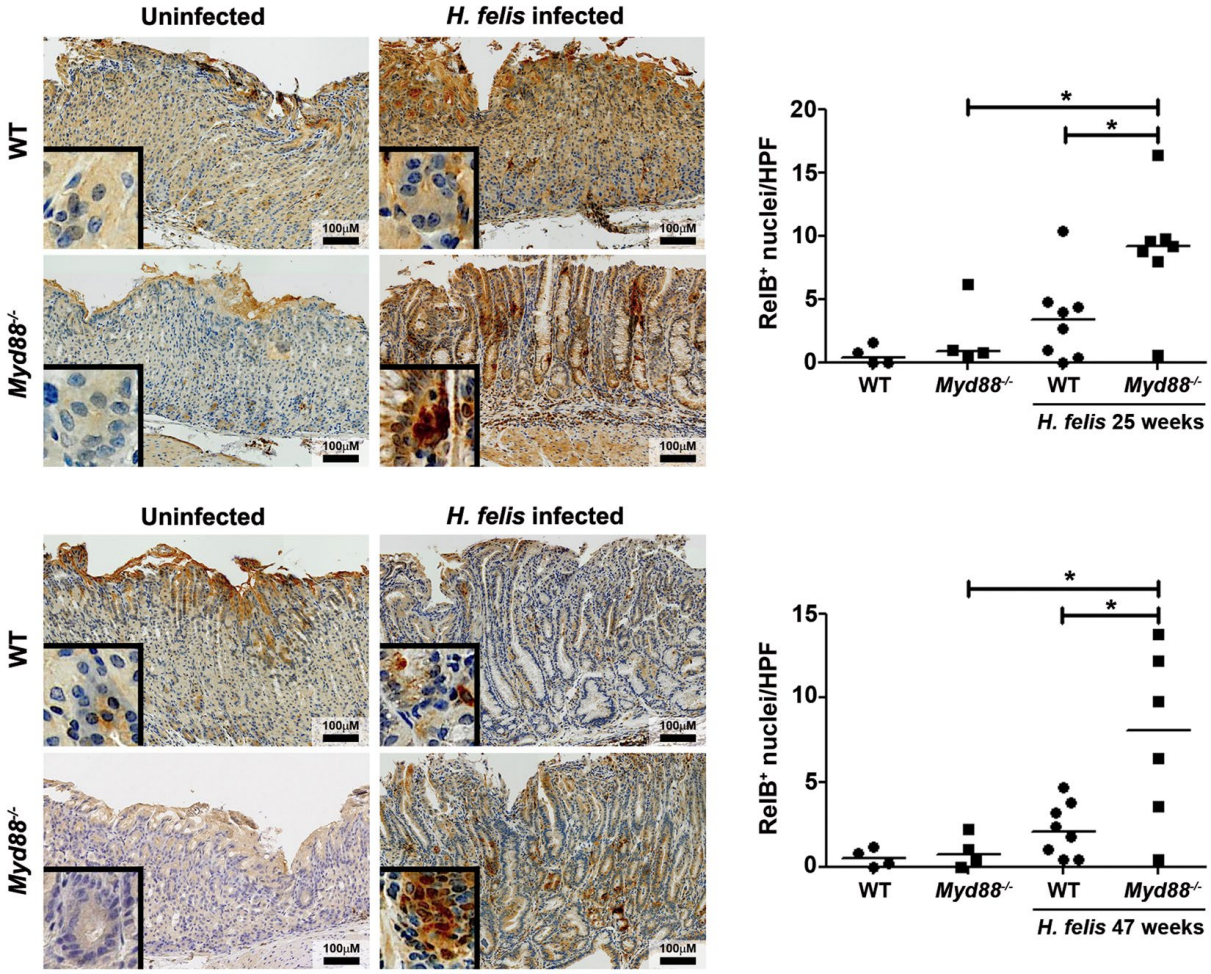
Non-canonical NF-κB signaling is enhanced in *Myd88*^−/−^ mice upon *H. felis* infection. Representative images of RelB expression in gastric tissue samples of wild type (WT) and *Myd88*^−/−^ mice control or infected with *H. felis* for 25 or 47 weeks. Quantification of RelB^+^ cells per high power field (HPF) (20x magnification) is shown. *p ≤ 0.05, **p ≤ 0.01. T-test. Each dot represents one mouse. Horizontal bars indicate medians.

Recently, *H. pylori* was reported to induce non-canonical NF-κB signaling in gastric epithelial cells, which highly contributed to the inflammatory response to the bacterium^10^. To assess whether non-canonical NF-κB signaling could be related to the more severe pathology observed in *Myd88*^−/−^ mice upon infection with *H. felis*, we first evaluated nuclear translocation of RelB into the nucleus of gastric epithelial cells. Mice infected with *H. felis* showed activation of non-canonical NF-κB (Fig. 1). Notably, mice deficient for MyD88 showed increased non-canonical NF-κB signaling in the stomach upon *H. felis* infection when compared to WT animals after 25 and 47 weeks of infection (Fig. 1). In contrast, there were no differences in activation of non-canonical NF-κB between WT and *Myd88*^−/−^ mice when left uninfected (Fig. 1). We confirmed activation of non-canonical NF-κB by staining the same tissue samples for NIK (Supplementary Fig. 1c). These observations suggest that, at least partially, enhanced activation of non-canonical NF-κB in response to *Helicobacter* infection might contribute to the more severe pathology observed in mice lacking MyD88.

### LIGHT might be the ligand responsible for non-canonical NF-κB activation upon H. felis infection

**Figure 2:**
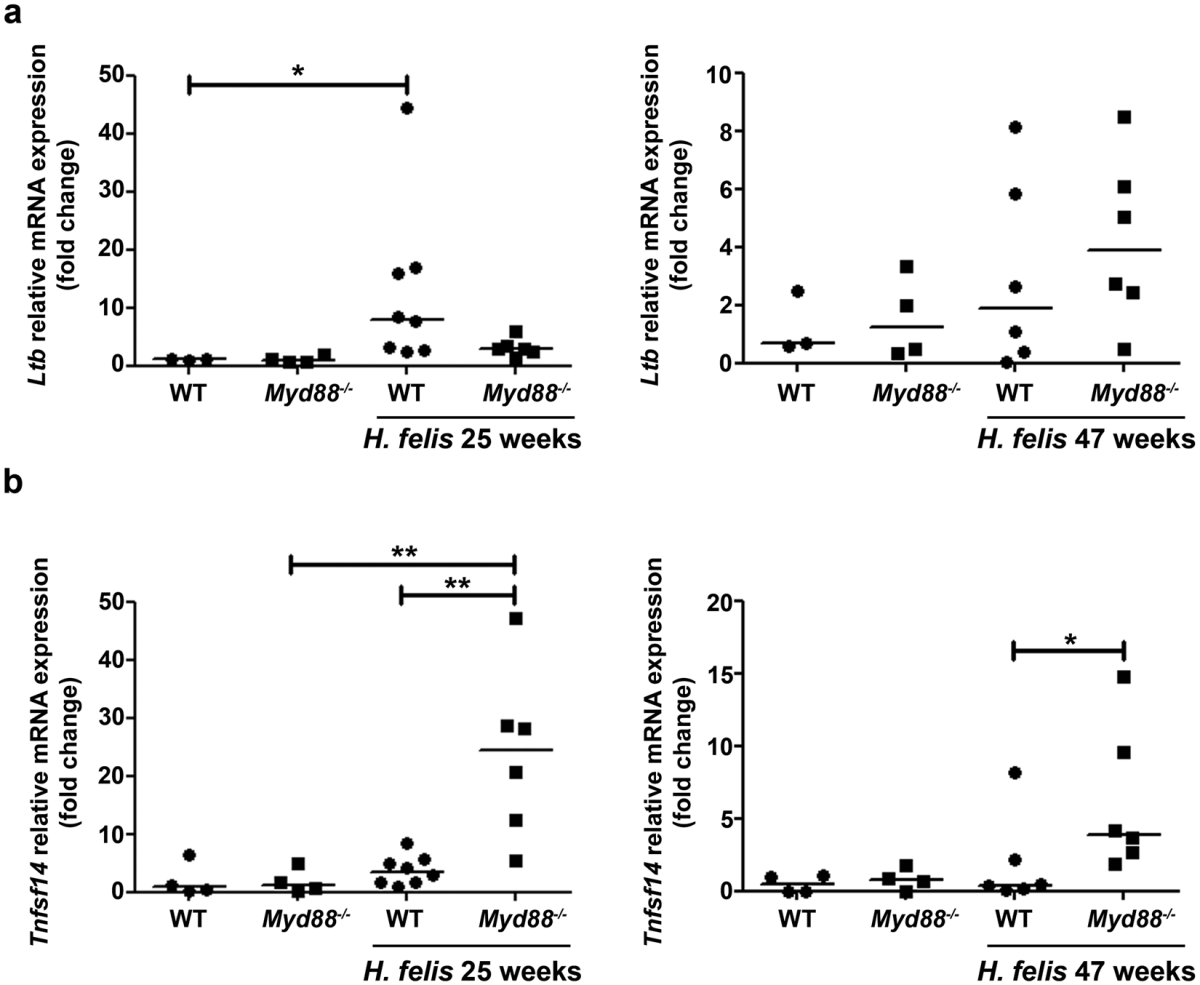
LIGHT is the main ligand inducing non-canonical NF-κB upon *H. felis* infection. Relative mRNA expression levels of *LTb* (**a**) and *Tnfsf14* (**b**) in the stomach of control and *H. felis-*infected mice at 25 and 47 weeks post-infection. Ct values were normalized to *Gadph*. *p ≤ 0.05, **p ≤ 0.01. Kruskal-Wallis test. Each dot represents one mouse. Horizontal bars indicate medians.

We recently showed that activation of non-canonical NF-κB by *H. pylori* was induced by LT through LTβR expressed on gastric epithelial cells^10^. Thus, we analyzed whether activation of non-canonical NF-κB signaling upon *H. felis* infection was driven by LT. Wild type- and *Myd88*^−/−^ infected mice showed increased levels of *Ltb* in the stomach (Fig. 2a). After 25 weeks we detected a significant increase in *Ltb* expression upon *H. felis* infection, which was not observed in *Myd88*^−/−^ infected mice. In addition, no differences in *Ltb* expression were detected between wild type and *Myd88*^−/−^ infected mice after 25 or 47 weeks (Fig. 2a), indicating that LTβ is not the ligand inducing non-canonical NF-κB activation during *H. felis* infection. Thus, we next analyzed *Tnfsf14*, which is also expressed in response to *H. pylori*^10^ and is an alternative ligand of LTβR activating non-canonical NF-κB. Notably, *H. felis* infection significantly increased *Tnfsf14* expression in the stomach of *Myd88*^−/−^ mice (Fig. 2b), while only a slight increase was detected in WT animals after 25 weeks of infection. We also performed immunohistochemistry for LIGHT, however the antibody used did not work reliably on formalin fixed, paraffin-embedded tissue sections. These results indicate that LIGHT is the main ligand activating non-canonical NF-κB signaling in response to *H. felis*.

### Lack of MyD88 relates to enhanced recruitment of T and B cells to the stomach upon H. felis infection

**Figure 3:**
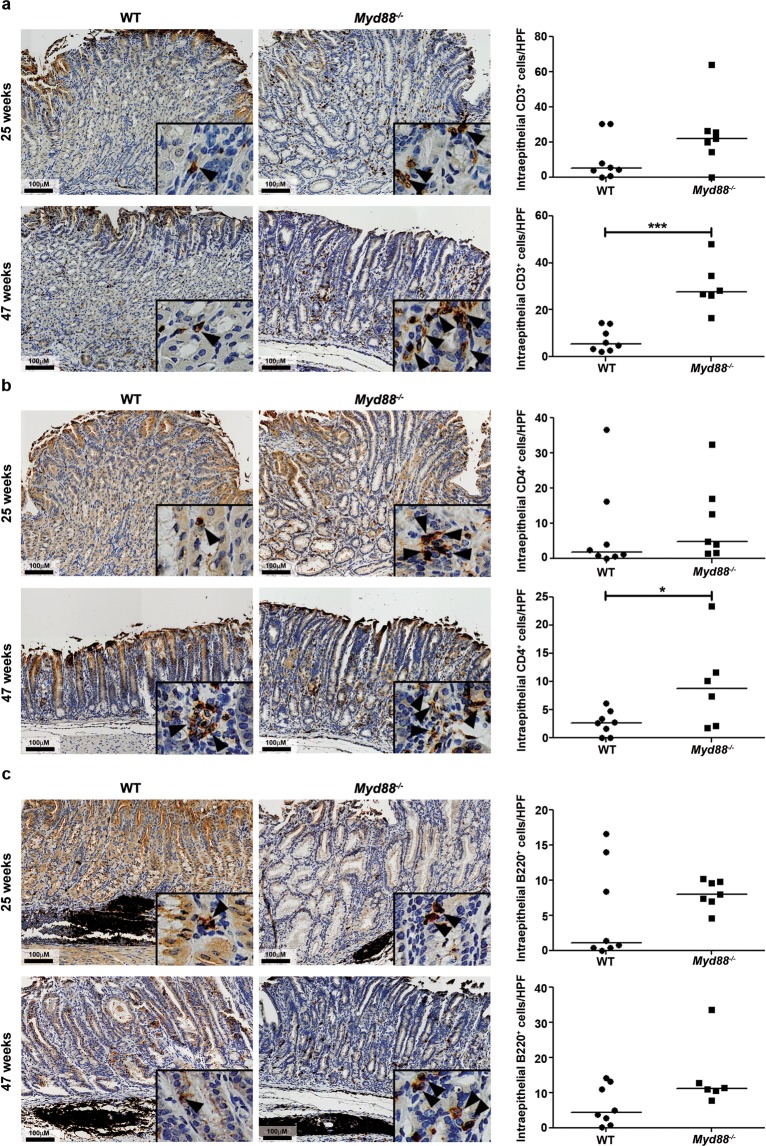
Recruitment of T and B lymphocytes into the stomach of *H. felis*-infected mice. Murine stomach samples of WT and *Myd88*^−/−^ mice infected with *H. felis* were stained for CD3 (**a**), CD4 (**b**), and B220 (**c**) by immunohistochemistry. Representative images are shown as well as quantification of CD3, CD4, and B220 positive cells (brown) per high power field (20x magnification). ***p ≤ 0.001. Mann-Whitney U test. Each dot represents one mouse. Horizontal bars indicate medians.

To analyze whether differential activation of non-canonical NF-κB pathway triggered by LIGHT translated into changes in the recruitment of inflammatory cells between WT and *Myd88*^−/−^ mice during *H. felis* infection, we stained stomach tissue samples for T cell, B cells, and macrophages. We observed that mice lacking MyD88 showed higher infiltration of CD3^+^ cells into the stomach when compared to WT mice (Fig. 3a). No differences in CD4^+^ cells recruitment was detected after 25 weeks of infection (Fig. 3b), while after 47 weeks of infection, higher numbers of CD4^+^ cells were observed in the gastric tissue of *Myd88*^−/−^ infected mice (Fig. 3b).

Wild type and *Myd88*^−/−^ mice showed large lymphoid structures in the gastric mucosa after infection (Fig. 3c). Those were mostly composed of B cells, which were also found infiltrating the epithelium. Notably, more intraepithelial B cells were detected in *Myd88*^−/−^ infected animals compared to WT mice (Fig. 3c), however the difference was not statistically significant.

No differences in macrophage infiltration between WT and *Myd88*^−/−^ mice after 25 or 47 weeks post-infection, as detected by F4/80 staining (Supplementary Fig. 2).

These results suggest that lack of MyD88 favors the recruitment of T cells into the gastric epithelium in response to *H. felis* infection.

### Cxcl9 and Icam1 are up-regulated in *Myd88*^−/−^ mice upon H. felis infection

We examined the expression of chemokines related to non-canonical NF-κB signaling that could explain the differences in T cell and B cell recruitment observed between WT and *Myd88*^−/−^ mice. We first analyzed the levels of *Cxcl13*, which is a B cell chemoattractant regulated by non-canonical NF-κB^6^. *H. felis* infection up-regulated *Cxcl13* expression in WT mice after 25 weeks of infection (Supplementary Fig. 3), while its expression was barely induced in *MyD88*^−/−^ infected mice (Supplementary Fig. 3). After 47 weeks, *Myd88*^−/−^ infected mice also showed up-regulation of *Cxcl13* expression in the stomach, albeit at a lower level when compared to WT mice (Supplementary Fig. 3). This observation suggests that *Cxcl13* might not be involved in the enhanced B cell recruitment observed in *Myd88*^−/−^ during *H. felis* infection.

**Figure 4:**
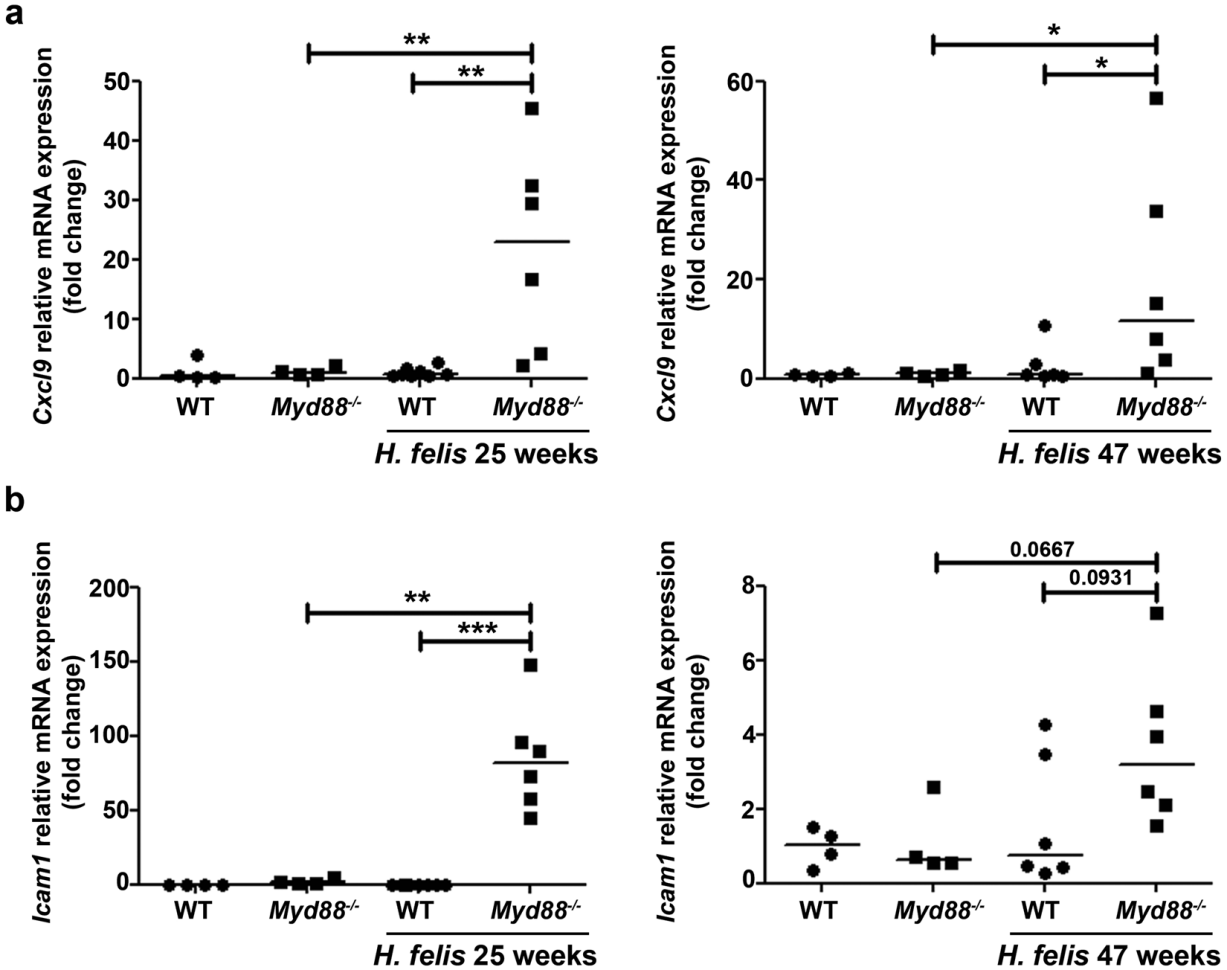
*Cxcl9* and *Icam1* are up-regulated in *Myd88*^−/−^ mice upon *H. felis* infection. *Cxcl9* (**a**) and *Icam1* (**b**) mRNA levels in control and *H. felis*-infected mice was determined by real-time PCR. Ct values were normalized to *Gadph*. *p ≤ 0.05, **p ≤ 0.01, ***p ≤ 0.001. Kruskal-Wallis test. Each dot represents one mouse. Horizontal bars indicate medians.

Next, we focused on chemokines involved in T cell recruitment. During *H. pylori* infection, *Cxcl9* was shown to contribute to the recruitment of inflammatory T cells into the gastric mucosa^15,16^. Interestingly this chemokine has been previously related to LIGHT and NF-κB signaling^6,17,18^. Infection with *H. felis* led to *Cxcl9* up-regulation only in *Myd88*^−/−^ mice (Fig. 4a) after 25 and 47 weeks of infection, while no induction was detected in WT animals.

Lastly, we analyzed the expression of *Icam1*, an adhesion molecule involved in transmigration of leucocytes into tissues, which can be regulated by non-canonical NF-κB^6^ and induced by LIGHT^19^. Similarly to *Cxcl9*, WT mice infected with *H. felis* did not show increased expression of *Icam1* in the stomach after 25 or 47 weeks of infection (Fig. 4b). In contrast, high levels of *Icam1* were detected in *Myd88*^−/−^ mice infected with *H. felis* for 25 weeks that were reduced but still high at 47 weeks post-infection (Fig. 4b). Together, these results suggest that CXCL-9 and ICAM-1, which have been previously reported to be induced by LIGHT, might be involved in the higher recruitment of T cells to the stomach of *Myd88*^−/−^ mice during *H. felis* infection.

### STAT3 activation is increased in *Myd88*^−/−^ mice upon H. felis infection

**Figure 5:**
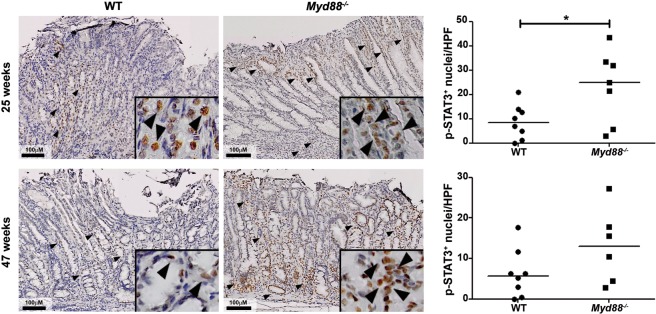
*H. felis* infection enhances STAT3 activation in *Myd88*^−/−^ mice. Representative images of p-STAT3 expression in the stomach of WT and *Myd88*^−/−^ mice infected with *H. felis* for 25 and 47 weeks. Quantification of p-STAT3^+^ cells per high power field (HPF) (20x magnification) is shown. *p ≤ 0.05. Mann-Whitney U test. Each dot represents one mouse. Horizontal bars indicate medians.

STAT3 signaling has been linked to severe gastric pathology during *H. pylori* infection as well as to gastric cancer development^3^. Interestingly, LIGHT was reported to activate STAT3 through non-canonical NF- κB signaling^14^. Therefore, we analyzed whether changes in STAT3 activation could correlate with *Tnfsf14* expression and the more severe pathology observed in *Myd88*^−/−^ mice. *H. felis* infection led to activation of STAT3 in the stomach of WT and *Myd88*^−/−^ mice after 25 and 47 weeks of infection (Fig. 5 and Supplementary Fig. 4). Notably, STAT3 activation was higher in *Myd88*^−/−^ mice, suggesting that activation of STAT3 might be linked to the gastric pathology observed in *Myd88*^−/−^ mice.

## Discussion

In a previous study we found MyD88 to play a protective role during *H. felis* infection^9^. Thus, mice deficient in MyD88 showed rapid progression of gastric pathology upon *H. felis* infection related to enhanced epithelial apoptosis and cell proliferation as well as altered expression of pro-inflammatory cytokines^9^. However, the molecular mechanisms driving early development of gastric malignancy in *Myd88*^−/−^ mice remained mostly elusive. Two recently published clinical data sets of human stomach cancers from The Cancer Genome Atlas (TCGA)^20,21^ revealed the presence of *MYD88* gene deletions and mutations in gastric and esophageal adenocarcinomas. These studies indicate for the first time a link between MyD88 deficiency and gastric cancer, thereby validating observations in *H. felis*-infected *Myd88*^−/−^ mice.

We recently showed that up-regulation of the ligands LT and LIGHT by *H. pylori* is responsible for activating non-canonical NF-κB in gastric epithelial cells through LTβR, driving the inflammatory response to *H. pylori*^10^. Therefore, we sought to investigate whether activation of non-canonical NF-κB could be related to the more severe pathology observed in mice deficient in MyD88. We observed up-regulation of *Ltb* expression upon *H. felis* infection but no differences between WT and *Myd88*^−/−^ mice that could explain the stronger activation of non-canonical NF-κB observed in the gastric epithelium of mice deficient for MyD88. In contrast, *Myd88*^−/−^ mice showed higher levels of *Tnfsf14* expression than WT mice, suggesting that LIGHT might be the ligand responsible for non-canonical NF-κB activation in these mice. What still remains unclear is how LIGHT is up-regulated in the context of *H. pylori or H. felis* infection and which *H. felis* bacterial factors can be involved, since *H. felis* lacks a type IV secretion system, which is important for *Tnfsf14* induction *in vivo* during *H. pylori* infection. Our previous observations indicated that during *H. pylori* infection *TNFSF14* expression was independent of canonical NF-κB activation^10^. Therefore, other signaling networks must be involved. In this context, regulation studies demonstrated an important role for Ca^2+^ signaling pathways and nuclear factor of activated T-cells (NFAT) transcription factors in LIGHT transcription induced by T cell activation. Moreover, Sp1 and Ets1 transcription factors were important for basal constitutive expression of LIGHT in T cells^22^. These pathways and transcription factors have been shown to be altered by *H. pylori*^23–25^ and thus might also be involved in LIGHT up-regulation. However, further experiments are needed to understand the mechanisms regulating LIGHT expression during *Helicobacter* infection.

The analysis of the gastric inflammatory infiltrates in *H. felis-*infected WT and *Myd88*^−/−^ mice showed increased recruitment of T cells as well as B cells in the stomach of mice lacking MyD88. Notably, *Tnfsf14* up-regulation in *Myd88*^−/−^ mice did not only correlate with hyperactivation of non-canonical NF-κB signaling, but also with the expression of chemokines (*Cxcl9*) involved in the recruitment of immune cells to sites of inflammation as well as with adhesion molecules (*Icam1*). Forced expression of LIGHT in tumors was found to increase the expression of interferon gamma (IFNγ) as well as chemoattractant cytokines including CXCL9^17^, while expression of ICAM1 was induced in bronchial epithelial cells upon treatment with LIGHT. Therefore, up-regulation of *Tnfsf14* elicited by *H. felis* infection in *Myd88*^−/−^ mice might contribute to immune cell recruitment by inducing the expression of chemoattractant molecules, specifically *Cxcl9*, since no differences in other chemokines such as *Cxcl10* or *Cxcl13* were detected between WT and *Myd88*^−/−^. Interestingly, CXCL9 was shown to contribute to the recruitment of inflammatory T cells into the gastric mucosa of *H. pylori* infected individuals^15^. In addition, *H. pylori* eradication in p27-deficient mice, which are prone to gastric cancer development upon infection, attenuated gastric pathology and this was associated with reduced expression of *Cxcl9* in the stomach^16^. Thus, LIGHT might support early and rapid progression of gastric pathology observed in *Myd88*^−/−^ mice by up-regulating the expression of chemokines important for the recruitment of inflammatory cells into the stomach.

Enhanced proliferation was detected in the gastric epithelium of *H. felis*-infected *Myd88*^−/−^ mice, contributing to the increased pathology observed in these mice^9^. Interestingly, interaction of LIGHT with LTβR was demonstrated to increase the survival and proliferation of bone marrow-derived mesenchymal stem cells through activation of survival and anti-apoptotic proteins^26^. In that study, activation of STAT3 by LIGHT played a central role by inducing the expression of platelet-derived growth factor (PDGF) and tumor growth factor β (TGFβ). We observed increased STAT3 activation in *Myd88*^−/−^ mice upon *H. felis* infection. The role of STAT3 in gastric carcinogenesis has been extensively reported. Hyperactivation of STAT3 in mice bearing a knock-in mutation in the gp130 receptor was sufficient to induce the development of gastric tumors^4^. In humans, STAT3 phosphorylation has been associated with worse prognosis of gastric tumors^27^ and several studies reported activation of STAT3 by *H. pylori* in gastric epithelial cells^12,28,29^. Notably, LIGHT-induced activation of STAT3 was shown to be mediated by NIK^14^, an essential kinase for the activation of non-canonical NF-κB. Thus, LIGHT can enhance its effects on cellular proliferation, survival, and inflammation by concomitant activation of non-canonical NF-κB and STAT3 signaling. Our results suggest that the activation of these two signaling pathways in mice lacking MyD88 might be a driving force for the development of gastric pathology induced upon *H. felis* infection.

Although our results suggest a link between pathology induced by *H. felis* and activation of non-canonical NF-κB, we cannot conclude whether our findings are secondary to *H. felis* infection or consequent upon the development of atrophy/dysplasia. Studying an earlier time point may help to elucidate this.

## Methods

### Mouse gastric tissue samples

Formalin-fixed, paraffin-embedded (FFPE) samples prepared from gastric samples from mice previously described in ref.^9^ were used in the present study. Briefly, 7 to 8-week old male mice in the C57BL/6 background, wild type (WT) and *Myd88* deficient (*Myd88*^−/−^) were infected with *H. felis* strain CS1 (ATCC 49179, Manassas VA) (8 mice per background) for 25 or 47 weeks^9,30–34^. Mice were inoculated with *H. felis* grown in Brain Heart Infusion (BHI) (10^9^ organisms/mouse) by oral gavage every other day for a total of 3 inoculations. Control mice received BHI only. During infection three *Myd88*^−/−^ mice died, one before and two after 25 weeks. At the selected time post-infection, mice were euthanized and stomachs were aseptically removed. Longitudinal cross-sections of gastric tissue from each mouse were cut, fixed in neutral buffered 10% formalin, and paraffin embedded. All *H. felis*-infected mice were confirmed colonized by assessing the flagella filament B (*flaB*) gene by real-time PCR, which was expressed similarly between WT and *Myd88*^−/−^ mice^9^. All animal procedures were performed in accordance with institutional guidelines and regulations and approved by the Animal Care Committee at the University of California, San Diego.

### Isolation of RNA from gastric tissue samples

RNA was isolated from FFPE gastric tissue samples using RecoverAll Total Nucleic Acid Isolation Kit (Life Technologies, Carlsbad CA) according to the manufacturer’s instructions. Briefly, a total of 20 pieces (20 μm thick/piece) of gastric tissue were cut using a microtome (Leica Biosystems, Buffalo Grove, IL). The gastric tissue sections were deparaffinilized with xylene followed by two washes in 100% ethanol to ensure complete paraffin removal and the pellet air dried. Protease digestion buffer was added to the pellet for 1 hr followed by RNA isolation buffer. The resulting RNA solution was passed through a filter cartridge and RNA eluted using nuclease-free water. RNA quality was determined using the Nanodrop system (Thermo Fisher, Waltham MA) by reading absorbance levels at 260 nm.

### Quantitative real-time RT-PCR

Real-time RT-PCR (reverse transcription polymerase chain reaction) was performed as previously described in our studies^9,35^ to determine expression of selected genes including, *Tnfsf14*, *Cxcl13*, *Cxcl9*, *Ltb*, and *Icam1*. RNA isolated from FFPE samples (2 μg/sample) was reverse transcribed into cDNA using High Capacity cDNA Reverse Transcription Kit (Thermo Fisher, Waltham MA). 2 μl of cDNA was used per well in a total of 10 μl reaction mix for amplification using the StepOne Real Time PCR (Applied Biosystems, Carlsbad CA). The amplification conditions consisted of an initial cycle of 95 °C for 5 minutes followed by 40 cycles of amplification with denaturation as follows: 95 °C for 15 sec, 60 °C for 20 sec, 72 °C for 40 sec. Gene expression levels were normalized to Glyceraldehyde 3-phosphate dehydrogenase (*Gapdh*). Data were analyzed using a comparative cycle threshold calculations (ΔΔC_T_, Applied Biosystems) and plotted using GraphPad Prism software. Primers used are listed in Supplementary Table 1.

### Immunohistochemistry

Gastric tissue samples from WT and *Myd88*^−/−^ control and *H. felis*-infected mice were incubated with specific antibodies overnight (Supplementary Table 2) after antigen retrieval in a pressure cooker using 0.01 M sodium citrate. These antibodies have been previously validated by our group and others^36–38^. After incubation with HRP-conjugated secondary antibodies, sections were developed using SignalStain DAB substrate (Cell Signaling) following manufacturer’s instructions. A secondary antibody only control was included to ensure that the secondary antibody did not unspecifically bind to certain cellular compartments. Samples were counterstained with hematoxylin (Morphisto). For automated image acquisition, Olympus Virtual Slide System VS120 (Olympus) was used. Stainings were visually quantified in a blinded manner. Five random high power field (HPF) areas per sample were scored blinded.

### Statistics

Statistical analysis was performed using GraphPad Prism. Normal distribution of the data was tested using Kolmogorov-Smirnov and D’Agostino & Pearson omnibus normality tests. Normal distributed data was analyzed using T-test, while non-normally distribute data were analyzed using Mann Whitney U Test. For multiple comparisons, ANOVA with Bonferroni’s correction (normal distribution) or Kruskal-Wallis with Dunn’s comparison test (non-normal distribution) were used. Statistical significance was established when p < 0.05.

## Supplementary information

Supplementary Material
