# Supplementary material for: Increased LIGHT expression and activation of non-canonical NF-κB are observed in gastric lesions of MyD88-deficient mice upon Helicobacter felis infection
Source: Sci Rep. 2019 May 7;9:7030. doi: 10.1038/s41598-019-43417-x (PMC6504916; doi:10.1038/s41598-019-43417-x)
Supplement: Supplementary file 1 — Supplementary Material [file 41598_2019_43417_MOESM1_ESM.pdf]

Increased LIGHT expression and activation of non-canonical NF-κB are observed in gastric lesions of MyD88-deficient mice upon *Helicobacter felis* infection

Raquel Mejías-Luque[1,2], Ivonne Lozano-Pope[3], Andreas Wanisch[1,2], Matthias Heikenwälder[4], Markus Gerhard[1,2] and Marygorret Obonyo[3*].

[1] Institut für Medizinische Mikrobiologie, Immunologie und Hygiene. Technische Universität München. Munich, Germany.

[2]German Centre for Infection Research (DZIF), partner site Munich; Munich; Germany.

[3] Department of Medicine, School of Medicine, University of California, San Diego, La Jolla, California, USA.

[4] Division of Chronic Inflammation and Cancer, German Cancer Research Center (DKFZ), Heidelberg, Germany.

*Correspondence: Marygorret Obonyo, mobonyo@ucsd.edu

Supplementary Figure legends

Supplementary Figure 1. *Myd88*$^{-/-}$ mice show early progression of gastric malignancy upon *H. felis* infection.

Representative images of hematoxylin-eosin (A), p65 (B) and NIK (C) stained tissue samples of wild type (WT) and *Myd88*$^{-/-}$ mice infected with *H. felis* for 25 or 47 weeks. Quantification of NIK positive cells is shown. Each dot represents one mouse. Horizontal bars indicate median.

Supplementary Figure 2. *Macrophage infiltration upon H. felis infection*

Murine stomach samples of WT and *Myd88*$^{-/-}$ mice infected with *H. felis* were stained for macrophages (F4/80) by immunohistochemistry. Representative images and quantification are shown. Each dot represents one mouse. Horizontal bars indicate median.

Supplementary Figure 3. *Cxcl13* mRNA levels upon *H. felis* infection

Cxcl13 mRNA levels in *H. felis*-infected mice was determined by real-time PCR. Ct values were normalized to GADPH. *p≤0.05, **p≤0.01. Kruskal-Wallis test. Each dot represents one mouse. Horizontal bars indicate median.

Supplementary Figure 4. *H. felis* infection enhances STAT3 activation in *Myd88*$^{-/-}$ mice

Representative images of p-STAT3 expression in the stomach of WT and *Myd88*$^{-/-}$ mice infected with *H. felis* for 25 and 47 weeks. Quantification of p-STAT3^{+} cells per high power field (HPF) (20x magnification) is shown. *p≤0.05. Kruskal-Wallis test. Each dot represents one mouse. Horizontal bars indicate median.

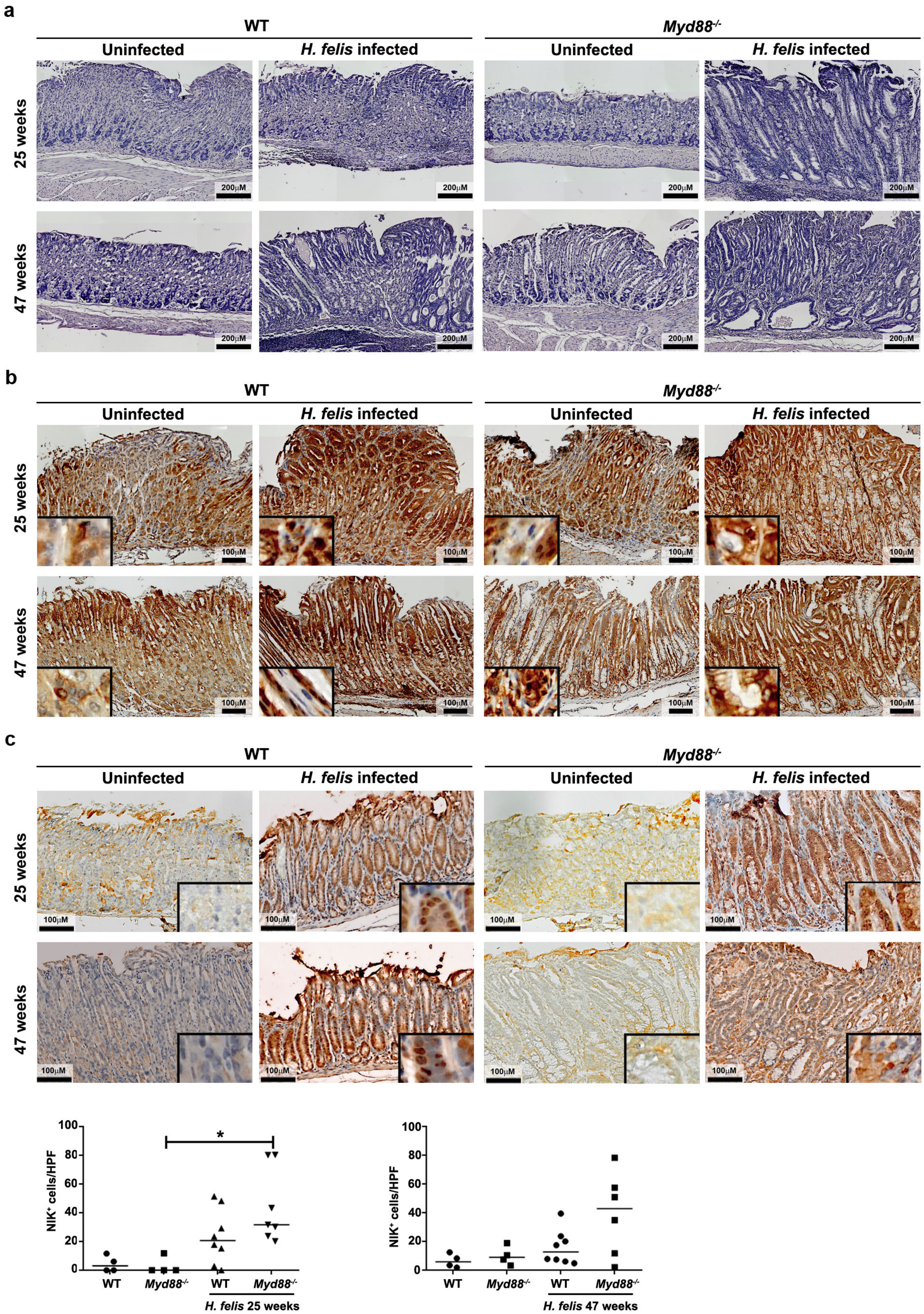
a
WT
Myd88-/-
Uninfected
H. felis infected
25 weeks
47 weeks
200μM
b
100μM
c
NIK+ cells/HPF
WT
Myd88-/-
H. felis 25 weeks
H. felis 47 weeks
*

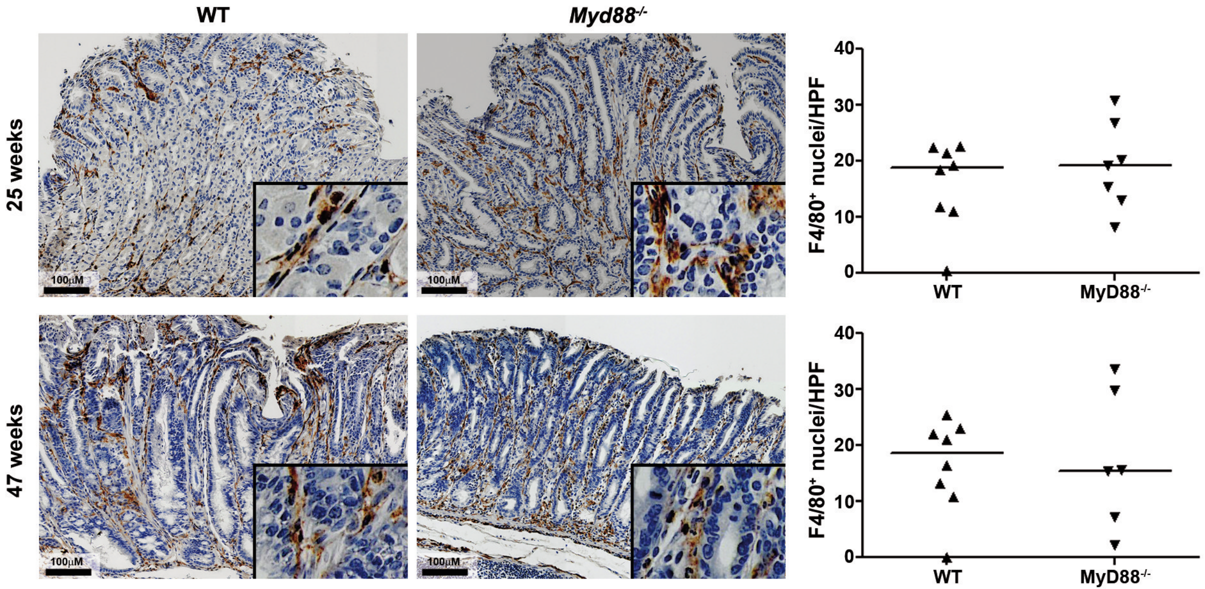
WT
Myd88-/-
25 weeks
47 weeks
100μM
100μM
100μM
100μM
F4/80+ nuclei/HPF
40
30
20
10
0
WT
MyD88-/-
F4/80+ nuclei/HPF
40
30
20
10
0
WT
MyD88-/-

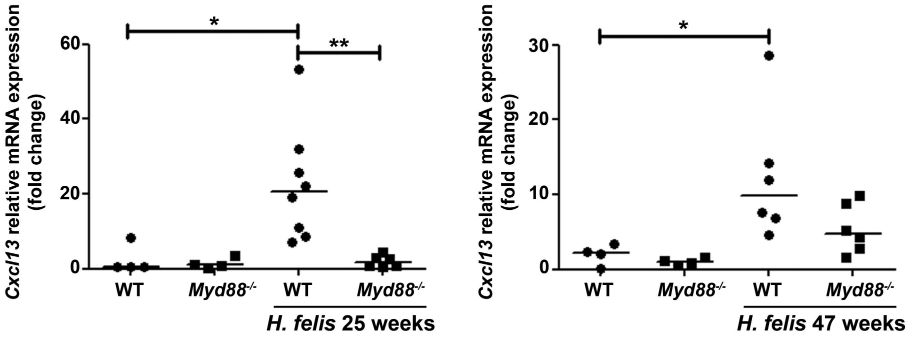

Cxcl13 relative mRNA expression (fold change)
60
40
20
0
*
**
WT
Myd88-/-
WT
Myd88-/-
H. felis 25 weeks
Cxcl13 relative mRNA expression (fold change)
30
20
10
0
*
WT
Myd88-/-
WT
Myd88-/-
H. felis 47 weeks

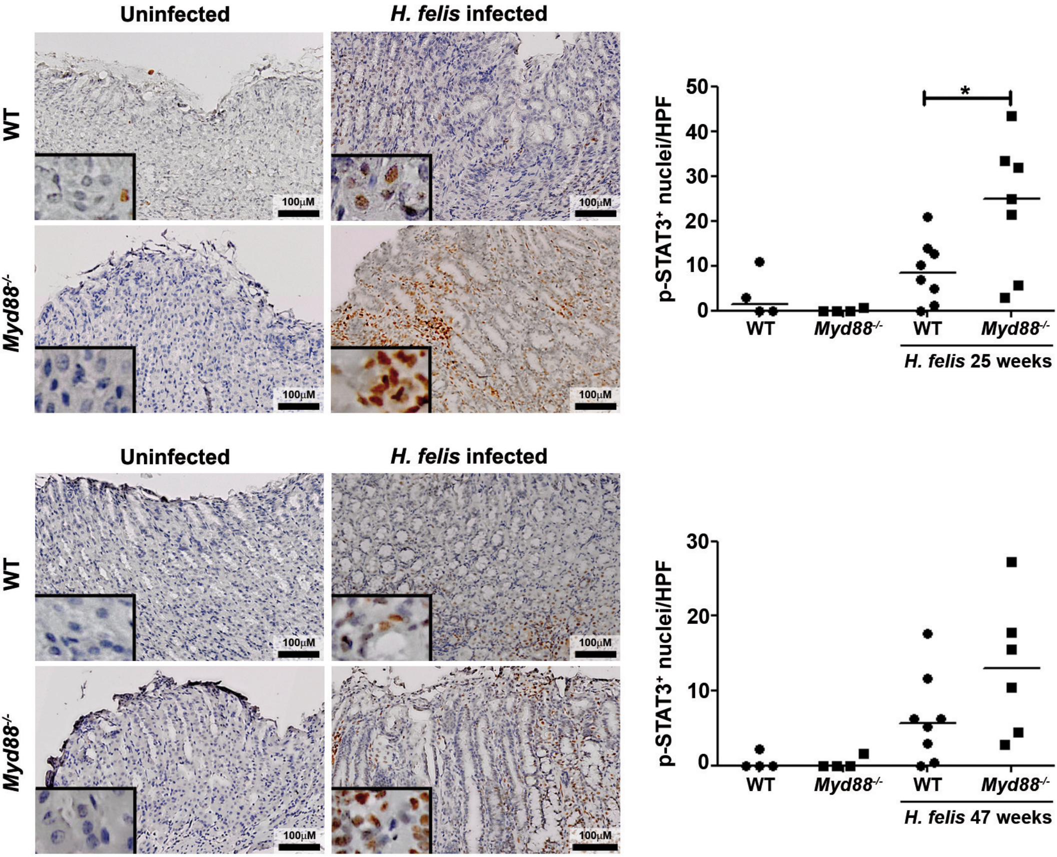
Uninfected
H. felis infected
WT
Myd88-/-
100µM
p-STAT3+ nuclei/HPF
50
40
30
20
10
0
*
WT
Myd88-/-
WT
Myd88-/-
H. felis 25 weeks
Uninfected
H. felis infected
WT
Myd88-/-
100µM
p-STAT3+ nuclei/HPF
30
20
10
0
WT
Myd88-/-
WT
Myd88-/-
H. felis 47 weeks

Supplementary Table 1: Primers Used for RT-PCR analysis:

Target	Sequence (5´-3´)	Reference
Gadph	F-TCAACAGCAACTCCCACTCTT CCA<br>R- ACCCTGTTGCTAGCCGTATTCA	1
Cxcl13	F- ATATGTGTGAATCCTCGTGCCA<br>R- GGGAGTTGAAGACAGACTTTTGC	2
Tnfsf14	F- TCCGCGTGCCTGG AAA<br>R- AAGCTCCGAAATAG GACCTGG	2
Ltb	F- TACACCAGATCCA GGGGTTC<br>R- ACTCATCCAAGCGC CTATGA	2
Icam1	F- CAATTTCTCATGCCGCACAG<br>R- AGCTGGAAGATCGAAAGTCCG	3
Cxcl9	F- GGAACCCTAGTGATAAGGAATGC<br>R- TGAGGTCTTTGAGGGATTTGTAGTG	4

1 Banerjee, A. *et al.* Deficiency of the myeloid differentiation primary response molecule MyD88 leads to an early and rapid development of Helicobacter-induced gastric malignancy. *Infection and immunity* **82**, 356-363, doi:10.1128/IAI.01344-13 (2014).

2 Mejias-Luque, R. *et al.* Lymphotoxin beta receptor signalling executes Helicobacter pylori-driven gastric inflammation in a T4SS-dependent manner. *Gut* **66**, 1369-1381, doi:10.1136/gutjnl-2015-310783 (2017).

3 Ren, G. *et al.* Inflammatory cytokine-induced intercellular adhesion molecule-1 and vascular cell adhesion molecule-1 in mesenchymal stem cells are critical for immunosuppression. *Journal of immunology* **184**, 2321-2328, doi:10.4049/jimmunol.0902023 (2010).

4 Wu, X., Lahiri, A., Haines, G. K., 3rd, Flavell, R. A. & Abraham, C. NOD2 regulates CXCR3-dependent CD8+ T cell accumulation in intestinal tissues with acute injury. *Journal of immunology* **192**, 3409-3418, doi:10.4049/jimmunol.1302436 (2014).

Supplementary Table 2: Antibodies used for Immunohistochemistry.

Target	Clone	Company
B220	RA3-6B2	BD Biosciences
CD3	SP7	NeoMarkers/Lab Vision Corporation
CD4	4SM95	eBioscience
F4/80	BM8	BMA Biomarkers
NF-kB p65	D14E12	Cell Signaling
RelB	C-19	Santa Cruz
p-STAT3	D3A7	Cell Signaling
NIK	-------	Biorbyt
